# Lowering *p*O_2_ Interacts with Photoperiod to Alter Physiological Performance of the Coastal Diatom *Thalassiosira pseudonana*

**DOI:** 10.3390/microorganisms9122541

**Published:** 2021-12-09

**Authors:** Bokun Chen, Jihua Liu, Ge Xu, Gang Li

**Affiliations:** 1 Institute of Marine Science and Technology, Shandong University, Qingdao 266237, China; chenbokunimst@163.com (B.C.); lddxnxysyb@163.com (G.X.); 2 Joint Laboratory for Ocean Research and Education of Dalhousie University, Shandong University and Xiamen University, Qingdao 266237, China; 3 Marine Environmental Monitoring Centre of Ningbo, East China Sea Bureau of Ministry of Natural Resources, Ningbo 315016, China; 4 Key Laboratory of Tropical Marine Bio-Resources and Ecology, South China Sea Institute of Oceanology, Chinese Academy of Sciences, Guangzhou 510530, China; ligang@scsio.ac.cn

**Keywords:** low *p*O_2_, photoperiod, photosynthesis, respiration, cell compositions, *Thalassiosira pseudonana*

## Abstract

Exacerbating deoxygenation is extensively affecting marine organisms, with no exception for phytoplankton. To probe these effects, we comparably explored the growth, cell compositions, photosynthesis, and transcriptome of a diatom *Thalassiosira pseudonana* under a matrix of *p*O_2_ levels and Light:Dark cycles at an optimal growth light. The growth rate (μ) of *T. pseudonana* under a 8:16 L:D cycle was enhanced by 34% by low *p*O_2_ but reduced by 22% by hypoxia. Under a 16:8 L:D cycle, however, the μ decreased with decreasing *p*O_2_ level. The cellular Chl *a* content decreased with decreasing *p*O_2_ under a 8:16 L:D cycle, whereas the protein content decreased under a 16:8 L:D cycle. The prolonged photoperiod reduced the Chl *a* but enhanced the protein contents. The lowered *p*O_2_ reduced the maximal PSII photochemical quantum yield (F_V_/F_M_), photosynthetic oxygen evolution rate (Pn), and respiration rate (Rd) under the 8:16 or 16:8 L:D cycles. Cellular malondialdehyde (MDA) content and superoxide dismutase (SOD) activity were higher under low *p*O_2_ than ambient *p*O_2_ or hypoxia. Moreover, the prolonged photoperiod reduced the F_V_/F_M_ and Pn among all three *p*O_2_ levels but enhanced the Rd, MDA, and SOD activity. Transcriptome data showed that most of 26 differentially expressed genes (DEGs) that mainly relate to photosynthesis, respiration, and metabolism were down-regulated by hypoxia, with varying expression degrees between the 8:16 and 16:8 L:D cycles. In addition, our results demonstrated that the positive or negative effect of lowering *p*O_2_ upon the growth of diatoms depends on the *p*O_2_ level and is mediated by the photoperiod.

## 1. Introduction

Anthropogenic marine eutrophication and warming are exacerbating the deoxygenation in the water column through, for example, unbalancing O_2_ production and consumption, lowering O_2_ solubility, and hindering the exchange with atmospheric O_2_ [1,2]. Over the past five decades, the global oceans have had an estimated loss of ~2% of their oxygen [3]; the degree and area of O_2_-deficiency or O_2_-limitation have extended quickly [1,4]. At present, the number of hypoxia zones (dissolved oxygen, DO < 2.5 mg L^−1^, ~25% saturation) have exceeded 500 worldwide, with the hypoxic area reaching over 1,000,000 km^2^ [4]. Dissolved O_2_ in seawater is important in marine ecosystems as the water must contain sufficient O_2_ to maintain aquatic biota. The reduced available O_2_ is known to threaten the health and lives of marine aerobic organisms that dwell in water or sediments, such as fish, shrimp, and shellfish, etc. [4,5]. Apart from affecting these aerobic organisms, the lowered available O_2_ also influences the photosynthetically O_2_ producing organisms [6,7,8], as these O_2_ producers, including phytoplankton, need surrounding O_2_ to maintain mitochondrial respiration under the limited-light or dark conditions, which can supply energy for cell growth and division [6] and even for survival [9]. Under sufficient light status, the produced O_2_ can compete with CO_2_ for the binding site of ribulose-1,5-bisphosphate carboxylase/oxygenase (RubisCO), which can bidirectionally catalyze the CO_2_ carboxylation in photosynthesis and O_2_ oxygenation in photorespiration [10]. The energy-costly CO_2_-concentrating mechanisms (CCMs) that are maintained in diatoms may improve the binding efficiency of CO_2_ over O_2_, through elevating the CO_2_ level around the binding site and tempering the unbalance between photosynthesis and photorespiration [11,12]. The decreased O_2_ may thus mechanically push the RubisCO-catalyzed biochemical reaction towards photosynthesis and increase the net primary production through suppressing photorespiration [13], although a case study showed that the lowered O_2_ reduced the growth of the diatom *Skeletonema costatum* [6]. 

In nature, phytoplankton live as deep as 200 m (euphotic zone) of the water column in the oceans and evolve various strategies to exist therein through, e.g., chain-forming and setae-own [14]. They can also be exposed occasionally or constantly to low O_2_ and even hypoxia that often vertically expands from thousands of meters to a few meters of seawater column [15]. The low O_2_/hypoxia phenomena even expands to shallower than 5 m in the Pearl River estuary [16] or less than 30 m in the Changjiang River estuary [17]. In these areas, phytoplankton cells usually vertically live from surface to deeper than 50 m depth [16,17]; they even live as deep as 150 m in the Arabia Sea, where the O_2_-deficient zone can expand to shallower than 50 m [15]. This means that most of phytoplankton cells in hypoxic areas experience a low O_2_/hypoxia status. Previous studies showed that lowered *p*O_2_ positively enhanced the growth of diatom *Thalassiosira pseudonana* [18] or Chlorophyta *Chlorella* spp. [19] and *Chlamydomonas reinhardtii* [20], while other studies showed that the lowered *p*O_2_ insignificantly affected the growth of *C. reinhardtii* [21] and even decreased the growth of diatom *S. costatum* [6], indicating the effects of lowering *p*O_2_ are still unclear. Other studies also referred to the survival of diatoms in anoxic conditions through using nitrate as an electron acceptor [22], as well as the contribution or function of the bloomed phytoplankton to the formation of hypoxic zones [23,24]. 

Dissolved O_2_ in seawater is one of the net products of phytoplankton if photosynthesis is over respiration under light status; while in the dark, this dissolved O_2_ is inversely consumed [25]. So, the duration of Light:Dark cycle (i.e., photoperiod), which often naturally varies with seasons or vertical mixings, may affect the physiological responses of phytoplankton to the surrounding O_2_ changes. Previously, the L:D cycle has been extensively observed to modulate cell growth and division, e.g., [26,27], as well as repair of photoinactivated PSII [28]. The L:D cycle interacting with light intensity or temperature has also been detected to alter cell compositions and antioxidant abilities in monospecies diatoms *Phaeodactylum tricornutum* [29], *T. pseudonan,* and *T. punctigera* [30,31], dinoflagellates *Alexnadrium minutum* and *A. catenella* [32,33], and *Chlorella vulgaris* [34], as well as in cell assemblies [35]. Nevertheless, it is unclear whether and, if so, how the L:D cycle interacts with reduced O_2_ to affect the physiology of phytoplankton.

Diatoms are important in marine ecosystems considering they account for ~20% of marine primary production [36] and sequester more carbon to the ocean’s interior with a fast sinking rate [14]. Diatoms are believed to dominate in global oceans, especially in estuarine or coastal waters where the lowered O_2_/hypoxia often occurs [2,4,16,23]. The genus *Thalassiosira* that appears in fresh-water, brackish, coastal, and oceanic environments are particular representatives [37]. Therefore, to explore the coupling effects of reduced O_2_ and photoperiod, we cultured a model diatom species *T. pseudonana* [38] under a matrix of pO_2_ level (ambient, 21% *v*/*v*; low *p*O_2_, 10%; and hypoxia, 3%) and Light:Dark cycle (L:D, 8:16 and L:D, 16:8) under an expected optimal growth instantaneous light intensity (150 μmol photons m^−2^ s^−1^) [30] and measured the growth, cell compositions, physiological performance, and transcriptome, which enabled us to answer how the lowered O_2_ interacts with the photoperiod to affect the coupling of growth and physiology of diatoms.

## 2. Materials and Methods

### 2.1. Culture Protocol and Growth Rate

A temperate marine centric diatom *T. pseudonana* (CCMP 1335), originally obtained from the Provasoli-Guillard National Center of Marine Phytoplankton (NCMP), was semi-continuously cultured with sterilized enriched artificial seawater (ESAW) in 500 mL conical flasks with 400 mL culture at 18 °C in an incubator (ZQZYCGF8, Shanghai, China). Light in the incubator was provided by a panel of LED (light-emitting diode) lamps and automatically turned on at 08:00 and turned off at 16:00 (8:16, Light:Dark cycle) or at 24:00 (16:8, L:D cycle). Light intensity was measured with a microspherical quantum sensor (QRT1, Hansatech, UK) in a culture flask filled with medium. Cultures were gently bubbled with the commercially prepared air (Qingdao Jinpeng Gas, China) with three *p*O_2_ levels [21% *v*/*v* (ambient *p*O_2_, Amb *p*O_2_), 10% *v*/*v* (Low *p*O_2_), and 3% *v*/*v* (Hypoxia)], which maintained the dissolved O_2_ (DO) in cultures at 310 ± 25, 150 ± 10, and 40 ± 7.5 μM, respectively. The DO concentration was monitored with an optode sensor controlled through Oxygen Logger software (OXR230, PyroScience Tech, Aachen, Germany). Before bubbling into cultures, the air streams filtered through a 0.2 µm microfilter were bubbled through another medium that was kept in the incubator, to eliminate the shock effects of varied *p*O_2_ and temperature during the medium replacement. No notable disruption of growth of *T. pseudonana* was detected by this bubbling when compared to no bubbling. In total, 18 separate semicontinuous cultures were grown under the combination (three replicates for each) of 3 *p*O_2_ levels and 2 photoperiods. Considering the seasonal photoperiod changes in temperate regions, we set the photoperiods at 8:16 and 16:8 L:D cycles, and we set the growth light at the approximately optimal intensity of 150 μmol photons m^−2^ s^−1^, according to [30,31].

The growth of *T. pseudonana* was estimated using chlorophyll *a* (Chl *a*) fluorescence with excitation of 440 nm and emission at 680 nm measured with a molecular device spectrofluorometer (CYT5M, BioTech, CA, USA) every morning (10:00 a.m., 2 h after lights on), before and after the dilutions with fresh medium. The Chl *a* in cultures was maintained at 0.86 ± 0.10 μg mL^−1^ during the cultivation period, and the specific growth rate (μ, d^−1^) was calculated as:

μ = [ln(N1) − ln(N0)]/(t1 − t0)

where N1 and N0 denote the OD680 value at time t1 and t0, respectively.

After the cultures went through over 9 generations, duplicate 5 mL cultures were taken at 10:00 a.m. from each flask after gently shaking and fixed with glutaraldehyde to a final concentration of 1%, for cell counting with an Accuri C6 flow cytometer (Becton-Dickinson, AZ, USA). After this, the aliquots of samples were taken for determination of cell compositions, physiological performance, and transcriptome as described below.

### 2.2. Chlorophyll Fluorescence

To measure the maximum photochemical quantum yield (F_V_/F_M_) of Photosystem II (PS II), 5 mL culture was taken from each flask and dark-acclimated for 15 min at growth temperature (18 °C). After this, the maximal (F_M_) chlorophyll fluorescence was measured with a fluorometer (AquaPen-C 100, Photon Systems Instruments, Prague, Czech Republic) under saturating blue-light pulse (3000 μmol photons m^−2^ s^−1^, 1 s), and the minimal fluorescence (F_O_) was measured in the presence of a weak modulated measuring light. The F_V_/F_M_ was calculated [39] as:

F_V_/F_M_ = (F_M_ − F_O_)/F_M_

Meanwhile, the relative electron transport rate (rETR) was measured 0, 10, 20, 50, 100, 300, and 500 μmol photon m^−2^ s^−1^ actinic lights, to obtain the rapid light curve (RLC) [40] as:

rETR = (F_M_’ − Ft)/F_M_’ × 0.5 × PAR

where F_M_’ and Ft are maximal and instantaneous fluorescence under each of 7 actinic lights (PAR, μmol photons m^−2^ s^−1^). The RLC-derived photosynthetic parameters, i.e., light utilization efficiency (α), maximal rETR (rETRmax), and saturation irradiance (E_K_, μmol photons m^−2^ s^−1^) were obtained [41] as:

rETR = PAR/(a × PAR^2^ + b × PAR + c)

α = 1/c, rETRmax = 1/[b + 2 × (a × c)^1/2^], E_K_ = c/[b + 2 × (a × c)^1/2^]

where a, b, and c are adjusted parameters.

### 2.3. Photosynthetic and Respiratory Rate

To measure the photosynthetic rate and dark respiratory rate of *T. pseudonana*, 300 mL cultures were collected from each of the combined *p*O_2_ and photoperiod treatments at the end of cultivation, followed by a 5-min dark-acclimation in a photosynthetic chamber where the temperature was maintained at 18 °C with a thermal cooler. Then, the increase in dissolved O_2_ concentration in the chamber under growth light and the decline in the dark were tracked with the optode oxygen sensor. The photosynthetic oxygen evolution rate (Pn) and respiratory rate (Rd, fmol O_2_ cell^−1^ min^−1^) were obtained by dividing the oxygen increase and decline rate by {cells mL^−1^ × measuring time}, respectively [31]. Each measurement lasted for ~15 min. After measuring the photosynthetic and respiratory rate, the culture was used for the following cell composition and transcriptome measurements.

### 2.4. Cell Compositions

To measure cellular Chl *a* concentration, a 50 mL culture from each treatment was vacuum filtered onto a Whatman GF/F glass fiber filter (25 mm in diameter), extracted in 4 mL 90% acetone (*v*/*v*) saturated with magnesium carbonate overnight at 4 °C in the dark. After 10 min centrifugation (10,000× *g*) at 4 °C, the optical absorption of the extraction was scanned with a spectrophotometer (Shimadzu model UV 1800-PC, Kyoto, Japan). Chl *a* concentration (μg mL^−1^) was calculated according to [42].

To measure protein concentration, a 50 mL culture was vacuum filtered onto a GF/F glass fiber filter and extracted in 1.0 mL pre-cooling buffer (pH 8.0, 20 mM Tris, 1 mM EDTA, 10 mM MgCl_2_, 50 mM NaHCO_3_, and 5 mM β-mercaptoethanol). The cells on filters were then broken through oscillating for 20 min with grinding beads at 4 °C using a vortex mixer (G560E, Scientific Industries, New York, NY, USA). After centrifugation (10,000× *g*, 10 min, 4 °C), the supernatant was used to quantify the protein using a protein assay kit (A045-2, Nanjing Jiancheng Biological Engineering Co., Nanjing, China) following the manufacturer’s protocol with the bicinchoninic acid (BCA) method [31,32]. The malondialdehyde (MDA) in the protein solution, a product of membrane lipid peroxidation, was determined using an assay kit (A003-1, Nanjing Jiancheng Biological Engineering Co., Nanjing, China) [32], as well as the superoxide dismutase (SOD) activity with an assay kit (A001-3, Nanjing Jiancheng Biological Engineering Co., Nanjing, China) [43] following the protocol of the manufacturer.

### 2.5. Transcriptome Sequencing and Analysis

At the end of cultivation, 80 mL cultures from each flask of each *p*O_2_ and photoperiod treatment were collected for transcriptome analysis. The total RNA of *T. pseudonana* was extracted with Trizol (Takara Bio. Inc., Shiga, Japan), and the degradation and purity of RNA were assessed by 1% agarose gels using a NanoPhotometer^®^ spectrophotometer (IMPLEN, Westlake Village, CA, USA) and an Agilent Bioanalyzer 2100 system (Agilent Tech., Santa Clara, CA, USA). Sequencing libraries were generated using NEBNext^®^ Ultra™ RNA Library Prep Kit for Illumina^®^ (NEB, BioLabs Inc., Ipswich, MA, USA). The library preparations were sequenced using the Illumina HiSeq platform to produce clean reads.

Transcriptome assembly was accomplished with the protocols of Trinity [44] and Corset [45]. Gene function was annotated on the base of following databases: Nr (NCBI non-redundant protein sequences), Nt (NCBI non-redundant nucleotide sequences), Pfam (Protein family), KOG/COG (Clusters of Orthologous Groups of proteins), Swiss-Prot (A manually annotated and reviewed protein sequence database), KO (KEGG Ortholog database), and GO (Gene Ontology). The RSEM (RNA-Seq by Expectation Maximization) was used to estimate the gene transcription levels [46]. Differential transcription analysis among the different *p*O_2_ and photoperiod treatments was performed with DEGseq package [47], and the p value was adjusted using the q-value, with the q-value of <0.005 and |log2(foldchange)| of >1 as significant threshold [48]. Metabolic pathway analysis of differentially expressed genes (DEGs) was conducted according to KEGG pathway database (http://www.genome.jp/kegg/, accessed on 10 November 2021), and functional enrichment analysis was performed using KOBAS (corrected *p* < 0.05) [49]. All the detailed information was supplied in supplemental materials.

### 2.6. Data Analysis

Data were shown as mean and standard deviations (mean ± sd). Paired *t*-test, one-way ANOVA with Tukey post-tests (Prism 5, Graphpad Software, San Diego, CA, USA), and comparisons of linear curve fits were used to detect the significant difference among cultures of each *p*O_2_ and photoperiod treatment. Two-way ANOVA with Tukey post-tests were used to detect the interactions of the *p*O_2_ level and photoperiod. The confidence level for the statistical tests was set at 0.05.

## 3. Results

**Figure 1:**
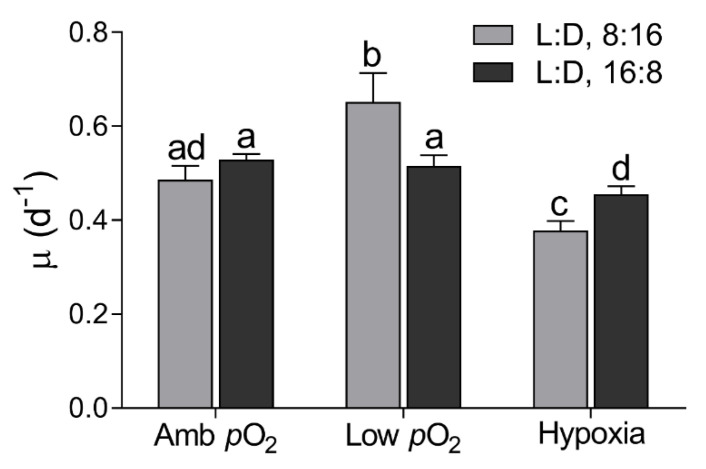
Specific growth rate (μ, day^−1^) of *T. pseudonana* under 8:16 and 16:8 Light:Dark (L:D) cycles, under ambient *p*O_2_ (Amb *p*O_2_), low *p*O_2_ (Low *p*O_2_), and hypoxia (Hypoxia) conditions. The vertical bar shows the standard deviation (*n* = 3), and different letters on top of the bar indicate the significant differences (*p* < 0.05, one-way ANOVA).

The growth of *T. pseudonana* under the light intensity of 150 μmol photonsm^−2^ s^−1^ differed greatly among different *p*O_2_ and photoperiod treatments (Figure 1). The specific growth rate (μ) was 0.49 ± 0.05 d^−1^ at ambient *p*O_2_, with no significant effect of photoperiod. The μ was enhanced by ~34% by low *p*O_2_ under the 8:16 L:D cycle but decreased by ~22% with further decreased *p*O_2_. Under the 16:8 L:D cycle, however, the μ decreased from 0.53 ± 0.02 to 0.46 ± 0.03 d^−1^ with decreasing *p*O_2_. Prolonged light duration did not affect the μ at ambient *p*O_2_ but enhanced it by ~21% at low *p*O_2_ and reduced it by ~20% at the hypoxia condition.

**Figure 2:**
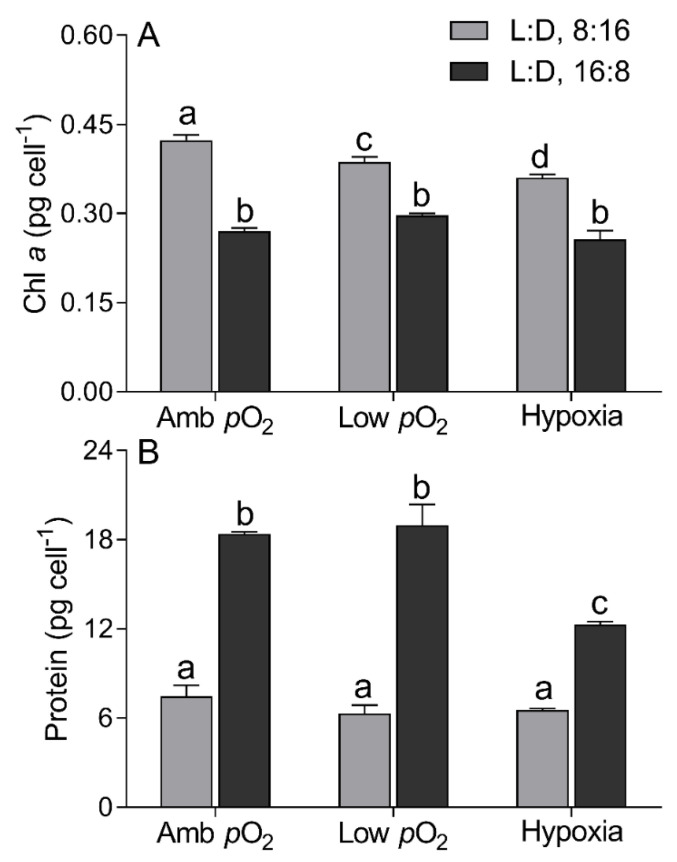
Chlorophyll *a* ((**A**), Chl *a*, pg cell^−1^) and protein contents ((**B**), pg cell^−1^) of *T. pseudonana* grown under 8:16 and 16:8 L:D cycles, under ambient *p*O_2_, low *p*O_2_, and hypoxia. The vertical bar shows the standard deviation (*n* = 3), and different letters on top of the bar indicate the significant differences (*p* < 0.05, one-way ANOVA).

Cellular Chl a content of *T. pseudonana* under the 8:16 L:D cycle decreased from 0.42 ± 0.02 to 0.36 ± 0.01 pg cell^−1^ from ambient *p*O_2_ to hypoxia conditions (Figure 2A). The prolonged light duration reduced the Chl *a* by ~36% at ambient *p*O_2_ and 23% and 29% at low *p*O_2_ and hypoxia, respectively. The protein content (6.78 ± 0.97 pg cell^−1^) under the 8:16 L:D cycle showed no significant difference among the three *p*O_2_ levels but decreased from 18.4 ± 0.25 to 12.3 ± 0.35 pg cell^−1^ from ambient *p*O_2_ to hypoxia (Figure 2B). The prolonged light duration enhanced the protein by ~150% at ambient *p*O_2_ and ~200% and ~87% at low *p*O_2_ or hypoxia, respectively.

**Figure 3:**
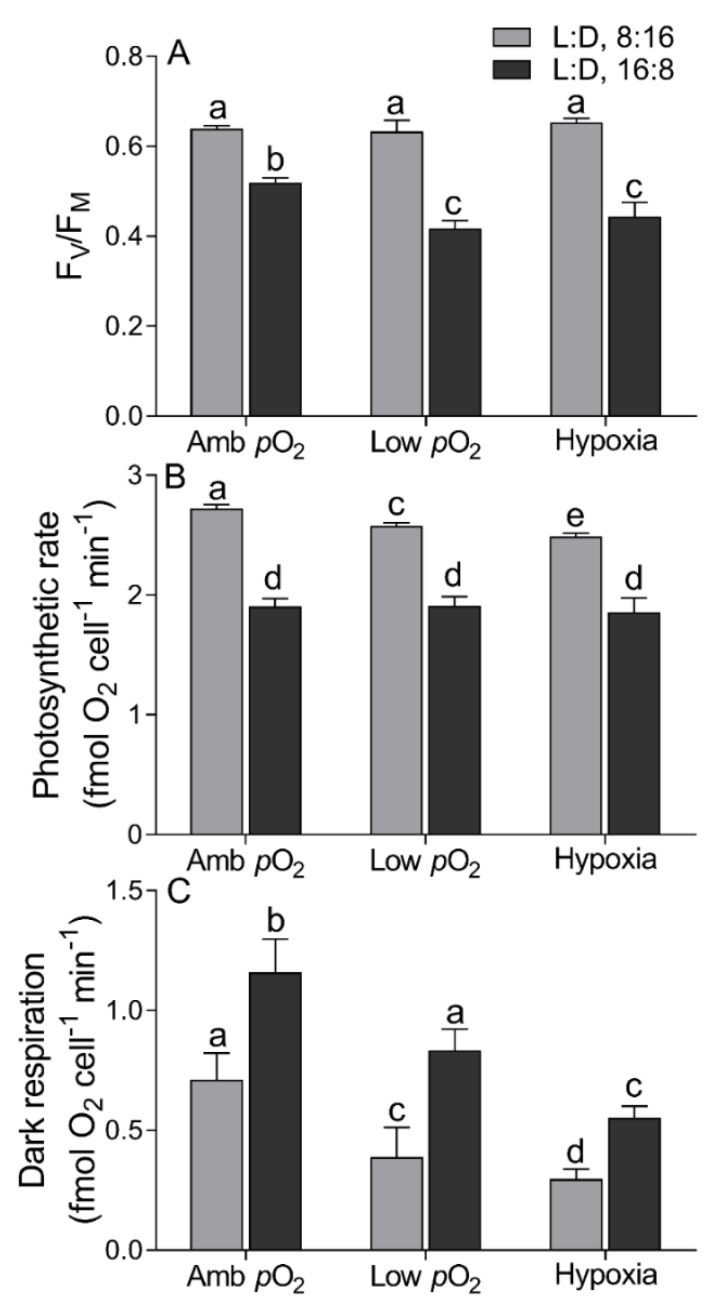
Maximal photochemical quantum yield of PSII ((**A**), F_V_/F_M_), photosynthetic oxygen evolution rate ((**B**), fmol O_2_ cell^−1^ min^−1^) and dark respiration rate ((**C**), fmol O_2_ cell^−1^ min^−1^) of *T. pseudonana* grown under 8:16 and 16:8 L:D cycles, under ambient *p*O_2_, low *p*O_2_, and hypoxia. The vertical bar shows the standard deviation (*n* = 3), and different letters on top of the bar indicate the significant differences (*p* < 0.05, one-way ANOVA).

The maximum PSII photochemical quantum yield (F_V_/F_M_) of *T. pseudonana* was 0.64 ± 0.02 under the 8:16 L:D cycle, with no significant effect of lowered *p*O_2_ (Figure 3A). Under the 16:8 L:D cycle, however, the F_V_/F_M_ decreased from 0.52 ± 0.02 to 0.42 ± 0.03 from ambient *p*O_2_ to hypoxia. The prolonged photoperiod reduced the F_V_/F_M_ by ~19% at ambient *p*O_2_ and by ~33% at low *p*O_2_ or hypoxia. In parallel, the RLC-derived light utilization efficiency (α) and maximal rETR (rETRmax) exhibited no significant difference among all three *p*O_2_ levels under both L:D 8:16 and 16:8 cycles; while the saturation irradiance (E_K_) was enhanced by low *p*O_2_ at the L:D 8:16 cycle (Table 1). The prolonged photoperiod significantly reduced the α, E_K_, and rETRmax among all *p*O_2_ treatments (*p* < 0.05) (Table 1). The photosynthetic oxygen evolution rate (Pn) under the 8:16 L:D cycle decreased from 2.72 ± 0.06 to 2.49 ± 0.05 fmol O_2_ cell^−1^ min^−1^ from ambient *p*O_2_ to hypoxia, while such a decreasing trend did not occur under the 16:8 L:D cycle (Figure 3B). The dark respiration rate (Rd) decreased from 0.71 ± 0.19 to 0.30 ± 0.07 fmol O_2_ cell^−1^ min^−1^ with decreasing *p*O_2_ under the 8:16 L:D cycle and from 1.16 ± 0.24 to 0.55 ± 0.08 fmol O_2_ cell^−1^ min^−1^ under the 16:8 L:D cycle (Figure 3C). The prolonged photoperiod significantly reduced the photosynthetic rate (*p* < 0.01) but enhanced the respiratory rate (*p* < 0.01) among all three *p*O_2_ treatments.

**Figure 4:**
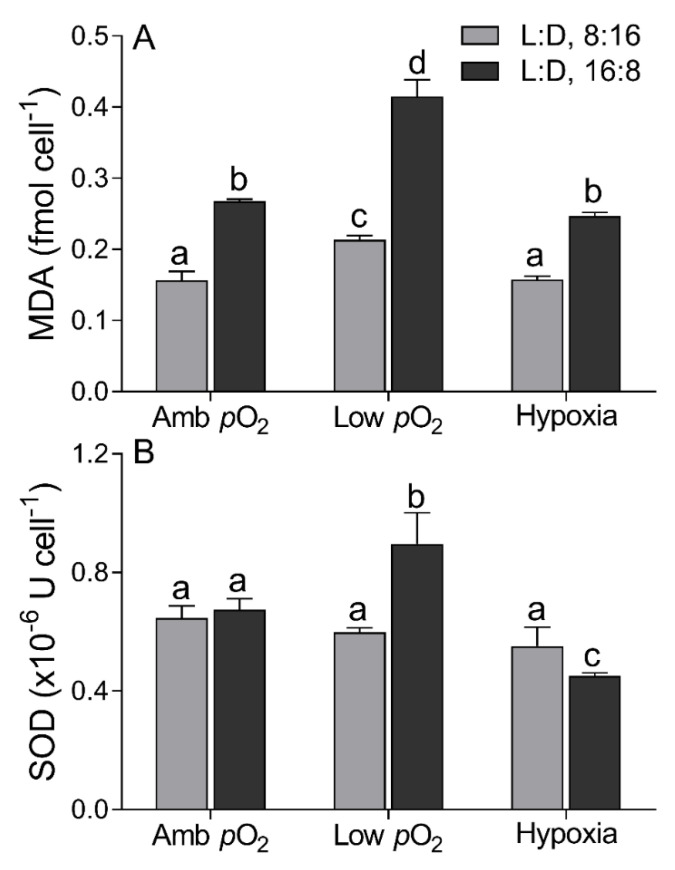
Cellular malondialdehyde concentration ((**A**), MDA, fmol cell^−1^) and superoxide dismutase activity ((**B**), SOD, ×10^−6^ U cell^−1^) of *T. pseudonana* grown under 8:16 and 16:8 L:D cycles, under ambient *p*O_2_, low *p*O_2_, and hypoxia. The vertical bar shows the standard deviation (*n* = 3), and different letters on top of the bar indicate the significant differences (*p* < 0.05, one-way ANOVA).

The cellular MDA content was 0.21 ± 0.01 fmol cell^−1^ at ambient *p*O_2_ under 8:16 L:D cycle, ~25% lower than that at low *p*O_2_ but similar to hypoxia (Figure 4A). The prolonged photoperiod enhanced the MDA by ~71%, ~95%, and ~56% at ambient *p*O_2_, low *p*O_2_, and hypoxia, respectively. The SOD activity was (0.60 ± 0.08) × 10^−6^ U cell^−1^ under 8:16 L:D cycle, with no significant effect of *p*O_2_ levels (Figure 4B). The prolonged photoperiod did not affect the SOD activity at ambient *p*O_2_ but enhanced it by ~50% at low *p*O_2_ and reduced it by ~18% at hypoxia.

**Figure 5:**
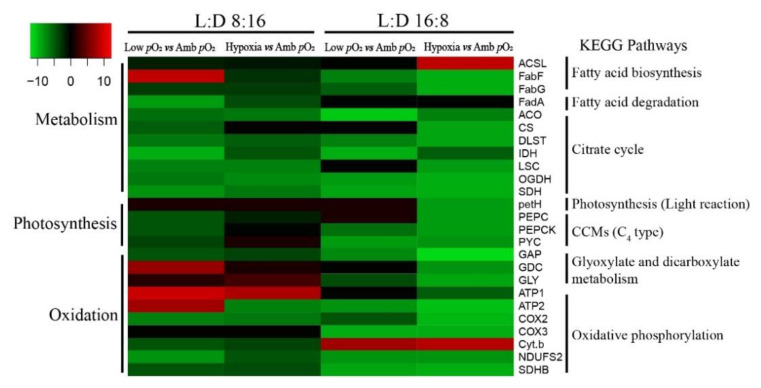
Heatmap of the low *p*O_2_ or hypoxia-caused changes (|log2(Fold Change)| > 2) in the differential expressed genes (DEGs) that are related to metabolism, photosystem, oxidation, and carbon fixation of *T. pseudonana* grown under 8:16 and 16:8 L:D cycles. Red and green colors indicate the up- and downregulations of the DEGs, respectively. The complete gene names are shown in Table S1.

**Figure 6:**
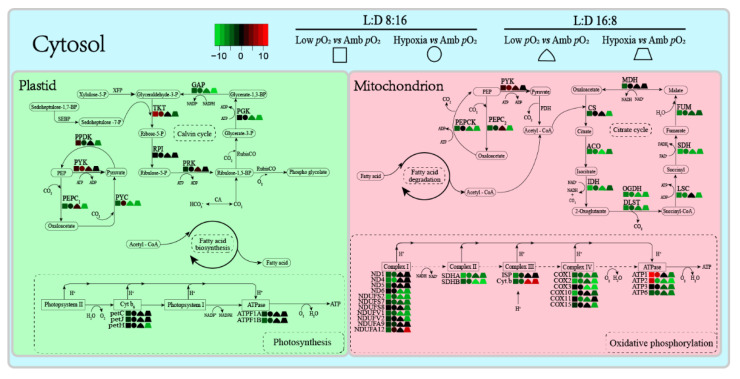
Schematic diagram of changed metabolic pathways of *T. pseudonana* under the varied *p*O_2_ and photoperiods. Red and green colors indicate the up- and downregulation of the DEGs, respectively. The solid arrow indicates the direct metabolic steps in a pathway. The complete gene names are shown in Table S1.

Transcriptome analysis showed that a total of 26 differentially expressed genes (DEGs) (|log2(foldchange)| > 8) that relate to photosynthesis, respiration, and metabolism were more sensitive to varying *p*O_2_ levels and photoperiods, based on the KEGG pathway enrichment (Table S1). Most of these identified DEGs were downregulated by lowered *p*O_2_ or hypoxia to varying degrees under the 8:16 and 16:8 L:D cycles, as compared to ambient *p*O_2_ (Figure 5). Of these DEGs, 11 and 7 transcripts were, respectively, related to the functions of metabolism (citrate cycle and fatty acid metabolism) and oxidation (oxidative phosphorylation), and 1 transcript was related to photosynthesis (light reaction). The transcriptional pattern seemed to be more sensitive to low *p*O_2_ in the citrate cycle, with 7 key nodal enzymes in four comparable strategies being downregulated (6.7-fold [avg.]) (Figure 6). Under the 16:8 L:D cycle, 7 DEGs of oxidative phosphorylation were downregulated by 6.3-fold [avg.] by low *p*O_2_ and by 4.5-fold [avg.] by hypoxia. Moreover, under the 8:16 L:D cycle, the expression of the ATP-targeted enzyme gene *ATP1* was upregulated by 9.6-fold and 8.1-fold by low *p*O_2_ or hypoxia, respectively, as compared to ambient *p*O_2_ (Figure S1, Table S1). The prolonged photoperiod mainly downregulated the expression of the photosynthesis-related genes, as well as the DEGs of the citrate cycle (Figure S1, Table S2). The downregulation of the RubisCO gene expression (1.47-fold [avg.]) by prolonged photoperiod was similar among all three *p*O_2_ treatments but different for that of C_4_ metabolism.

## 4. Discussion

Most previous studies reported the individual effect upon phytoplankton physiology by lowering *p*O_2_ levels [6,7,18] or varying photoperiods [30,31,32,33,34]. In this study, we showed the coupling effects of lowered *p*O_2_ and photoperiods on the physiological performance of the diatom *T. pseudonana*. Lowering *p*O_2_ interacted with longer photoperiod to reduce the light-harvesting pigments and photosynthetic rate through downregulating the genes related to photosynthesis and metabolism and, consequently, reduced the growth of *T. pseudonana* (F_(2,12)_ = 6.46, *p* < 0.05). Moreover, at lowered *p*O_2_, the reduction in respiration and photosynthesis occurred under both the 8:16 and 16:8 L:D cycles, as compared to ambient *p*O_2_; while the enhancement of the growth rate was present under the former but reduced under the latter L:D cycle, which indicated the alteration of light duration on the balance of the lowered *p*O_2_-induced savings of consumption and the reduction in photosynthetic production.

Longer light duration reduced the cellular pigment content of *T. pseudonana* (Figure 2A). Similar to previous studies [30,31], the light intensity of 150 µmol photons m^−2^ s^−1^ was expected to saturate the cell growth under a shorter photoperiod and may have oversaturated under the longer photoperiod, as indicated by an insignificant difference of the µ between the 8:16 and 16:8 L:D cycles (Figure 1). It is easily understood that phytoplankton cells usually lower the synthesis and accumulation of Chl *a* in oversaturated light, to lessen the excessive energy harvesting [43] and thus to alleviate the excessive light energy-caused photodamage or photoinhibition [30,50]. Moreover, the decreased *p*O_2_ interacted with the prolonged photoperiod to aggravate the decrease in Chl a (F_(2,12)_ = 7.53, *p* < 0.01), as also supported by the transcriptome results (Figure 6). This can be explained by the fact that the decreased *p*O_2_ has pulled the RubisCO-catalyzed biochemical reaction towards the photosynthetic process [13] thus saving the energy derived from the light-harvesting complex. Such energy savings may feedback to adaptively cause cells to lower the capacity for light harvesting through lowering the Chl *a* containing photosystems. If considering the function of CCM for concentrating CO_2_ in diatoms [10,11,12], however, the positive effect of reduced *p*O_2_ may be tempered as indicated by the reduction in growth in the diatom *T. pseudonana* at hypoxia condition (Figure 1) or in *Skeletonema costatum* [6] and *C. reinhardtii* [21]. Furthermore, an increase in cellular proteins occurred under the combined hypoxia and longer photoperiod status (Figure 2B), which may help to further save energy; more cellular proteins usually indicate more enzymes and, thus, more activity of biochemical reactions within cells [10,13,51].

The longer photoperiod suppressed the photosynthetic efficiency of *T. pseudonana* but enhanced the respiration (Figure 3), indicating the growth light here was oversaturated under the 18:6 photoperiod. Such an adverse effect was aggravated by the extremely low *p*O_2_ (F_V_/F_M_, F_(2,12)_ = 4.14, *p* < 0.05; and Pn, F_(2,12)_ = 3.65, *p* < 0.05), consistent with more products of membrane lipid peroxidation, i.e., higher cellular MDA content at hypoxia status (Figure 4A). Under such an adverse circumstance, phytoplankton cells often adaptively promote the ROS-scavenged ability, including enhancing cellular SOD activity to protect themselves against oxidative damage [52]. Our findings supported this with higher SOD activity at low *p*O_2_ (Figure 4B); at hypoxia, however, the SOD activity was depressed, suggesting the cellular O_2_ level was too low to maintain the ROS-scavenging capacity, thus, leading to the enhancement of MDA content, although the lowered *p*O_2_ circumstance theoretically goes against the ROS production and MDA accumulation [53]. Supporting the decrease in photosynthetic capacity, the transcriptome results indicated the downregulation of most DEGs, including those related to the photosynthetic process (Figure 6). Compared to photosynthesis, the lowered *p*O_2_ reduced the respiration even more, leading to an even lower respiration rate at hypoxia (Figure 3C), supported by the severe downregulation of DEGs related to the key enzymes in the citric acid cycle (Figure 5 and Figure 6). Such downregulation also occurred in *Chlorella vulgaris* that had overcome the metabolic constraint through lowering respiration and allocating more fixed C to maintain growth [54], as well as in *T. pseudonana* at a low *p*O_2_ state (Figure 1 and Figure 3C). Mechanically, the dissolved O_2_ most likely affects the ATP synthesis in mitochondria [55] and RubisCO-catalyzed photorespiration in plastid [56]. It is the case in this study, as the decreased *p*O_2_ downregulated a total of 25 genes that relate to ATP synthesis (Figure 6), which may have lowered the biochemical activities within cells through inhibiting, e.g., *PPDK* [57] and, thus, reduced the respiration rate (Figure 3C). Moreover, the expression of C_4_ genes such as *PEPC*, *PPDK,* and *PEPCK*, showed no linear correlation with decreasing *p*O_2_ (Figure S1), consistent with previous results [11,12]. Finally, the expressions of *GAP*, *GDC,* and *GLY* genes that relate to photorespiration were also downregulated by lowering *p*O_2_ (Figure 6), as well as the decreased photosynthetic efficiency, the mechanical contradiction of which needs to be studied further.

## 5. Conclusions

In this study, we found the hypoxia and longer light period interactively reduced the growth of diatom *T. pseudonana* through reducing the photosynthetic capacity by downregulating the related genes’ expression. Growing under lowered *p*O_2_ condition, both cellular Chl *a* and protein contents were lower, but the MDA content and SOD activity were higher and the lowered *p*O_2_-induced effect was mediated by the light duration. Moreover, our results demonstrated that whether there is a reduction in respiration by the lowered *p*O_2_ over that of photosynthesis or not determines the positive and negative effects of lowering *p*O_2_ on the growth of diatoms, which depends on the *p*O_2_ level and is mediated by the photoperiod.

## Figures and Tables

**Table 1:** The rapid light curve (RLC)-derived light utilization efficiency (α), saturation irradiance (E_K_, μmol photons m^−2^ s^−1^), and maximal relative electron transport rate (rETRmax) of *T. pseudonana* grown under 8:16 and 16:8 Light:Dark cycles, under ambient *p*O_2_ (Amb *p*O_2_), low *p*O_2_, and hypoxia.

Photosynthetic Parameters	L:D 8:16			L:D 8:16		
	Amb *p*O_2_	Low *p*O_2_	Hypoxia	Amb *p*O_2_	Low *p*O_2_	Hypoxia
*α*	0.32 ± 0.01 ^a^	0.31 ± 0.02 ^a^	0.31 ± 0.04 ^a^	0.25 ± 0.01 ^b^	0.19 ± 0.04 ^b^	0.25 ± 0.02 ^b^
*E*_K_	469 ± 52.5 ^a^	582 ± 63.6 ^b^	499 ± 46.1 ^a^	200 ± 52.3 ^c^	230 ± 43.8 ^c^	230 ± 42.0 ^c^
rETR_max_	148 ± 14.2 ^a^	179 ± 39.4 ^a^	159 ± 46.4 ^a^	49.7 ± 15.3 ^b^	43.8 ± 11.8 ^b^	57.1 ± 14.3 ^b^

Note: Numbers indicate the mean and standard deviation from measurements of 3 independent cultures (*n* = 3); and different letters at the top-right of the numbers indicate significant differences (*p* < 0.05, one-way ANOVA).

## Data Availability

The data presented in this study are available within the article.

## Supplementary Materials

The following are available online at https://www.mdpi.com/article/10.3390/microorganisms9122541/s1, Figure S1: Heatmap of the photoperiod-caused changes (|log2(Fold Change)| > 2) in the DEGs related to metabolism, photosystem, oxidation, and carbon fixation *of T. pseudonana* under ambient *p*O_2_, low *p*O_2_, and hypoxia; Table S1: The log-2-fold value of the DEGs of *T. pseudonana* regulated by lowered *p*O_2_ under 8:16 and 16:8 L:D cycles, relevant to respiration, photosynthesis, carbon fixation, and metabolism.
