# Supplementary material for: Lowering pO2 Interacts with Photoperiod to Alter Physiological Performance of the Coastal Diatom Thalassiosira pseudonana
Source: Microorganisms. 2021 Dec 9;9(12):2541. doi: 10.3390/microorganisms9122541 (PMC8704836; doi:10.3390/microorganisms9122541)
Supplement: Supplementary file 1 [file microorganisms-09-02541-s001.zip › microorganisms-1479987-supplementary.pdf]

Supplemental materials

Figure S1: Heatmap of the photoperiod-caused changes (|log2(Fold Change)|>2) in the differential expressed genes (DEGs) related to metabolism, photosystem, oxidation and carbon fixation of *T. pseudonana* under ambient pO_2 (Amb pO_2), low pO_2 (Low pO_2) and hypoxia (Hypoxia). Red and green colors indicate the up- and down-regulations of the DEGs, respectively. The complete gene names are shown in Table S2.

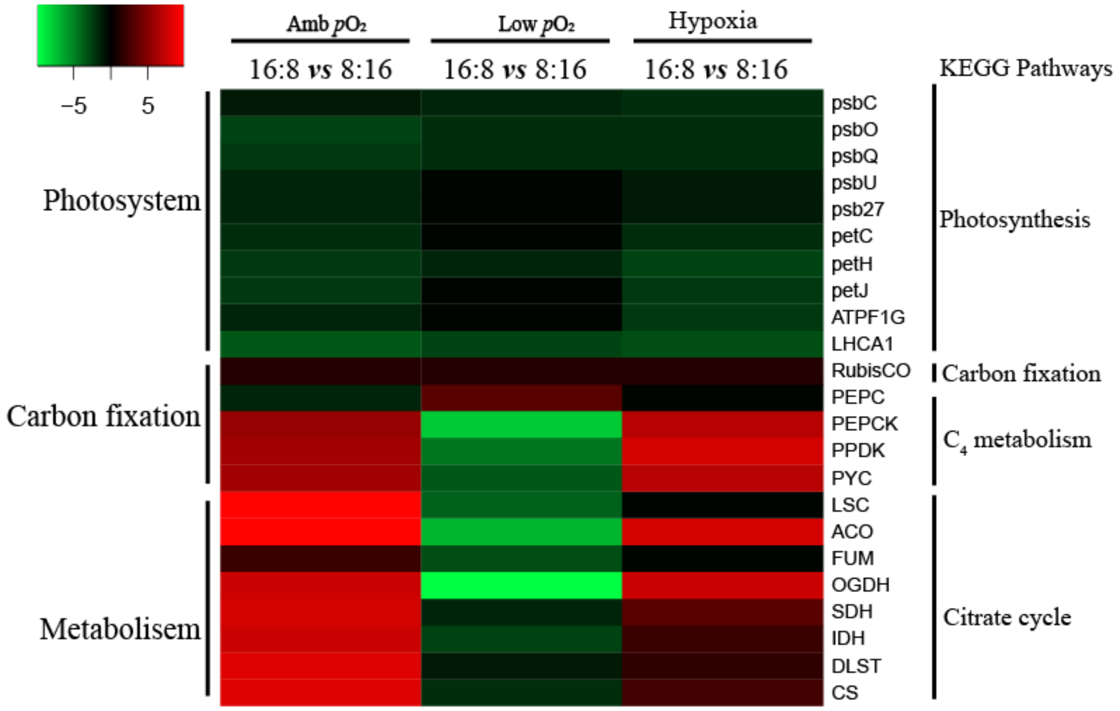

Table S1: The log-2-fold value of DEGs of *T. pseudonana* regulated by lowered pO_2 under 8:16 and 16:8 L:D cycles, that are relevant to respiration, photosynthesis, carbon fixation and metabolism.

	L:D 8:16		L:D 16:8			
Name	Low pO_2 *vs* Amb pO_2	Hypoxia *vs* Amb pO_2	Low pO_2 *vs* Amb pO_2	Hypoxia *vs* Amb pO_2	Complete name	KEGG pathway
TKT	6.41	4.56	0.00	-4.72	transketolase	Calvin cycle
ALDO	-1.38	0.00	0.00	7.08	fructose-bisphosphate aldolase	
SEBP	0.00	0.00	0.00	0.00	sedoheptulose-1,7-bisphosphatase	
XFP	0.00	0.00	0.00	0.00	xylulose-5-phosphate/fructose-6-phosphate phosphoketolase	
RPI	-1.06	0.00	0.00	0.00	ribose 5-phosphate isomerase	
GAP	0.00	1.67	0.00	0.00	glyceraldehyde-3-phosphate dehydrogenase (NADP+)	
TPI	-4.54	-2.36	0.00	0.00	triosephosphate isomerase	
GPI	0.00	0.00	0.00	0.00	glucose-6-phosphate isomerase	
PRK	-1.38	0.00	1.20	0.00	phosphoribulokinase	
FBP	0.00	0.00	0.00	0.00	fructose-1,6-bisphosphatase	
G6PC	0.00	0.00	0.00	0.00	glucose-6-phosphatase	
Rubisco	0.00	0.00	0.00	0.00	ribulose-1,5-bisphosphate carboxylase/oxygenase	
PGK	0.00	0.00	-7.20	-7.86	phosphoglycerate kinase	
PPDK	1.22	-2.74		-7.12	pyruvate, orthophosphate dikinase	C_4 metabolism
PEPC	-4.60	-1.52	1.16	-8.63	phosphoenolpyruvate carboxylase	

PYC	-3.73	1.19	-8.69	-8.18	pyruvate carboxylase	
PYK	2.42	4.48	1.29		pyruvate kinase	
PEPCK	-4.45	0.00	-5.96	-8.47	phosphoenolpyruvate carboxykinase (ATP)	
PGAM	6.70	1.68	0.00	0.00	2,3-bisphosphoglycerate-dependent phosphoglycerate mutase	
ENO	-5.89	1.85	0.00	0.00	enolase	
PGP	0.00	0.00	0.00	0.00	phosphoglycolate phosphatase	glyoxylate and dicarboxylate metabolism
GLC	0.00	2.08	-7.34	-7.46	glycolate oxidase	
CAT	0.00	0.00	0.00	0.00	catalase	
GLY	1.79	3.04	-3.94	-9.10	glycine hydroxymethyltransferase	
GDC	7.21	1.36	0.00	-8.09	glycine dehydrogenase	
GGAT	1.34	1.60	0.00	0.00	glutamate--glyoxylate aminotransferase	
AGXT	0.00	0.00	0.00	0.00	alanine-glyoxylate transaminase	
GHR	0.00	0.00	0.00	-7.62	glyoxylate/hydroxypyruvate reductase	
GLYK	0.00	0.00	0.00	0.00	D-glycerate 3-kinase	
GLU	0.00	1.61	-2.62	-7.80	glutamate synthase (ferredoxin)	
GLN	6.85	2.50	-2.46	-5.56	glutamine synthetase	
G6PD	0.00	-1.43	-2.84	-7.42	glucose-6-phosphate 1-dehydrogenase	Pentose phosphate pathway
PGL	0.00	0.00	0.00	0.00	6-phosphogluconolactonase	
6PGD	0.00	0.00	0.00	0.00	6-phosphogluconate	

					dehydrogenase	
RPE	0.00	0.00	0.00	0.00	ribulose-phosphate 3-epimerase	
CS	-4.87	0.00	0.00	-8.83	citrate synthase	
ACO	-5.99	-6.69	-11.06	-7.06	aconitate hydratase	
IDH	-9.48	-4.83	-9.27	-4.94	isocitrate dehydrogenase	
DLST	-6.37	-5.15	-7.12	-8.82	2-oxoglutarate dehydrogenase	
LSC	-6.81	-6.88	0.00	-8.63	succinyl-CoA synthetase	
SDH	-7.97	-6.33	-9.13	-9.25	succinate dehydrogenase (ubiquinone)	Citrate cycle
FUM	-6.11	-4.92	-4.28	-5.40	fumarate hydratase	
NAD-ME	-5.21	0.00	0.00	0.00	malate dehydrogenase (oxaloacetate-decarboxylating) (NADP+)	
OGDH	-6.39	-7.65	-8.31	-9.23	2-oxoglutarate dehydrogenase E1 component	
PSB27	-1.19	0.00	0.00	0.00	photosystem II Psb27 protein	
PETC	-1.19	0.00	0.00	0.00	cytochrome b6-f complex iron-sulfur subunit	
PETJ	-1.59	0.00	0.00	0.00	cytochrome c6	
petH	0.00	0.00	0.00	-8.85	ferredoxin--NADP+ reductase	
ATPF1B	-3.78	-1.90	0.00	0.00	F-type H+/Na+-transporting ATPase subunit beta	Photosynthesis
ATPF1A	-1.33	0.00	0.00	0.00	F-type H+/Na+-transporting ATPase subunit alpha	
LHCA1	-3.48	-2.64	0.00	-1.01	light-harvesting complex I chlorophyll a/b binding protein 1	

ACAC	-7.57	-7.36	7.98	-7.55	acetyl-CoA carboxylase	Fatty acid biosynthesis
FabF	9.15	-2.53	-7.25	-9.51	3-oxoacyl-[acyl-carrier-protein] synthase II	
FabG	-3.36	-3.02	-5.04	-9.61	3-oxoacyl-[acyl-carrier protein] reductase	
FabI	-4.15	-2.08	-7.34	7.47	enoyl-[acyl-carrier protein] reductase I	
FabZ	-1.21	0.00	0.00	0.00	3-hydroxyacyl-[acyl-carrier-protein] dehydratase	
ACSL	-1.55	-1.06	0.00	-9.02	long-chain acyl-CoA synthetase	
FabH	6.77	0.00	3.78	-4.33	3-oxoacyl-[acyl-carrier-protein] synthase III	
FabD	0.00	0.00	0.00	-6.96	[acyl-carrier-protein] S-malonyltransferase	
SOD	-5.53	0.00	-8.99	-9.11	superoxide dismutase	Peroxisome
ACSL	-1.55	-1.06	0.00	-9.02	long-chain acyl-CoA synthetase	Fatty acid degradation
ACOX	0.00	0.00	0.00	-4.60	acyl-CoA oxidase	
GCDH	0.00	0.00	3.26	-7.55	glutaryl-CoA dehydrogenase	
ACAT	-6.72	-3.55	-5.35	-5.77	acetyl-CoA C-acetyltransferase	
ALDH	-6.37	-2.34	0.00	-5.60	aldehyde dehydrogenase (NAD+)	
HADH	-4.89	-2.43	0.00	0.00	3-hydroxyacyl-CoA dehydrogenase	
FadA	-8.49	-4.22	0.00	0.00	acetyl-CoA acyltransferase	
ND1	-4.37	-3.19	0.00	0.00	NADH-ubiquinone oxidoreductase chain 1	Oxidative phosphorylation

ND4	-6.51	-7.76	0.00	0.00	NADH-ubiquinone oxidoreductase chain 4
ND5	-3.80	0.00	0.00	0.00	NADH-ubiquinone oxidoreductase chain 5
ND6	0.00	0.00	-7.57	-7.71	NADH-ubiquinone oxidoreductase chain 6
NDUFS2	-8.03	-4.41	-7.96	-8.09	NADH dehydrogenase (ubiquinone) Fe-S protein 2
NDUFS7	-2.59	-3.76	-4.20	-5.23	NADH dehydrogenase (ubiquinone) Fe-S protein 7
NDUFS8	0.00	0.00	0.00	-6.71	NADH dehydrogenase (ubiquinone) Fe-S protein 8
NDUFV1	-4.03	-3.83	-7.63	-7.76	NADH dehydrogenase (ubiquinone) flavoprotein 1
NDUFV2	-1.06	0.00	0.00	-7.65	NADH dehydrogenase (ubiquinone) flavoprotein 2
NDUFA9	-1.53	-1.11	0.00	0.00	NADH dehydrogenase (ubiquinone) 1 alpha subcomplex subunit 9
NDUFA12	-4.14	1.68	0.00	7.79	NADH dehydrogenase (ubiquinone) 1 alpha subcomplex subunit 12
SDHA	-7.97	-6.33	-4.65	-5.98	succinate dehydrogenase (ubiquinone) flavoprotein subunit
SDHB	-4.74	-3.96	-9.13	-9.25	succinate dehydrogenase

					(ubiquinone) iron-sulfur subunit
ISP	-5.03	-2.70	0.00	0.00	ubiquinol-cytochrome c reductase iron-sulfur subunit
Cyt b	-4.42	-3.56	7.52	8.43	ubiquinol-cytochrome c reductase cytochrome b/c1 subunit
COX10	-7.46	0.00	0.00	-3.84	heme o synthase
COX1	-4.48	-3.81	-5.76	-6.69	cytochrome c oxidase subunit 1
COX2	-6.85	-6.64	-4.62	-10.0	cytochrome c oxidase subunit 2
COX3	0.00	0.00	-9.40	-9.53	cytochrome c oxidase subunit 3
COX11	0.00	-4.88	-6.67	0.00	cytochrome c oxidase assembly protein subunit 11
COX15	-1.07	0.00	0.00	-6.93	heme a synthase
ATP1	9.63	8.10	0.00	-4.87	F-type H+-transporting ATPase subunit alpha
ATP2	7.61	-6.88	-7.90	-10.65	F-type H+-transporting ATPase subunit beta
ATP3	0.00	0.00	-3.06	-5.78	F-type H+-transporting ATPase subunit gamma
ATP6	-4.46	-4.21	-4.51	-5.95	F-type H+-transporting ATPase subunit a

Table S2: The log-2-fold value (n=2) of DEGs of *T. pseudonana* regulated by photoperiod under ambient pO_2 (Amb pO_2), low pO_2 (Low pO_2) and hypoxia (Hypoxia), that are relevant to respiration, photosynthesis, carbon fixation and metabolism.

Name	16:8 L:D *vs* 8:16 L:D Amb pO_2	Low pO_2	Hypoxia	Complete name	KEGG pathway
RubisCO	1.47	1.51	1.45	ribulose-1,5-bisphosphate carboxylase/oxygenase	Calvin cycle
psbC	-1.12	-1.2	-1.67	photosystem II CP43 chlorophyll apoprotein	Photosynthesis
psbO	-2.38	-1.92	-1.85	photosystem II oxygen-evolving enhancer protein 1	
psbQ	-2.21	-1.69	-1.86	photosystem II oxygen-evolving enhancer protein 3	
psbU	-1.31	0	-1.11	photosystem II PsbU protein	
psb27	-1.5	0	-1.12	photosystem II Psb27 protein	
petC	-1.69	0	-1.62	cytochrome b6-f complex iron-sulfur subunit	
petH	-2.06	-1.42	-2.74	ferredoxin--NADP+ reductase	
petJ	-2.3	0	-2.26	cytochrome c6	
ATPF1G	-1.46	0	-2.01	F-type H+-transporting ATPase subunit gamma	
LHCA1	-3.5	-2.61	-2.95	light-harvesting complex I chlorophyll a/b binding protein 1	
PEPC	-1.54	3.17	0	phosphoenolpyruvate carboxylase	C4 metabolism
PEPCK	5.69	-7.48	6.79	phosphoenolpyruvate carboxykinase (ATP)	
PPDK	5.88	-4.56	7.92	pyruvate, orthophosphate dikinase	
PYC	5.88	-3.25	6.75	pyruvate carboxylase	
LSC	9.45	-3.76	0	succinyl-CoA synthetase	Citrate cycle
ACO	9.49	-6.97	8.16	aconitate hydratase	
FUM	2.22	-2.81	0	fumarate hydratase	
OGDH	7.61	-9.78	7.68	2-oxoglutarate dehydrogenase E1 component	
SDH	7.93	-1.26	3.24	succinate dehydrogenase (ubiquinone)	
IDH	7.61	-2.66	1.98	isocitrate dehydrogenase	
DLST	8.3	-1.02	1.96	2-oxoglutarate dehydrogenase	
CS	8.31	-1.95	2.37	citrate synthase	

Table S3: Sequencing results of the transcriptome data of *T. pseudonana* under 8:16 and 16:8 Light:Dark (L:D) cycles, under ambient pO_2 (Amb pO_2), low pO_2 (Low pO_2) and hypoxia (Hypoxia) conditions.

Light cycle	Sample	Clean Reads	Clean Bases	Error (%)	Q20 (%)	Q30 (%)	GC Content (%)
L:D 8:16	Amb pO_2 1	20982710	6.29G	0.03	97.3	92.68	48.32
	Amb pO_2 2	21031757	6.31G	0.03	97.23	92.61	48.19
	Low pO_2 1	22018345	6.61G	0.03	97.26	92.53	48.42
	Low pO_2 2	21253450	6.38G	0.03	97.27	92.58	48.13
	Hypoxia 1	21215167	6.36G	0.03	97.38	92.72	49.06
	Hypoxia 2	22068261	6.62G	0.03	97.39	92.88	48.73
L:D 16:8	Amb pO_2 1	22034592	6.61G	0.03	96.57	91.56	46.51
	Amb pO_2 2	21943845	6.58G	0.03	96.32	91.14	45.48
	Low pO_2 1	21462414	6.44G	0.03	97.01	91.95	47.28
	Low pO_2 2	21426806	6.43G	0.03	96.91	91.99	46.3
	Hypoxia 1	21673292	6.50G	0.03	97.55	93.1	48.06
	Hypoxia 2	21424291	6.43G	0.03	97.37	92.7	47.89

Table S4: Numbers of unigenes annotated in seven databases of *T. pseudonana* grown under 8:16 and 16:8 Light:Dark (L:D) cycles, under ambient pO_2 (Amb pO_2), low pO_2 (Low pO_2) and hypoxia (Hypoxia) conditions.

	Number of Unigenes	Percentage (%)
Annotated in NR	19249	62.24
Annotated in NT	8672	28.04
Annotated in KO	7181	23.22
Annotated in SwissProt	17397	56.25
Annotated in PFAM	19390	62.7
Annotated in GO	19389	62.7
Annotated in KOG	6845	22.13
Annotated in all Databases	2267	7.33
Annotated in at least one Database	24945	80.66
Total Unigenes	30923	100

Table S5: Statistical results of DEGs of *T. pseudonana* grown under 8:16 and 16:8 Light:Dark (L:D) cycles, under ambient pO_2 (Amb pO_2), low pO_2 (Low pO_2) and hypoxia (Hypoxia) conditions.

Light cycle	O_2 concentration	All	Up	Down
L:D 8:16	Low pO_2 *vs* Amb pO_2	3048	1397	1651
	Hypoxia *vs* Amb pO_2	2333	1160	1173
	Low pO_2 *vs* Hypoxia	2343	1216	1127
L:D 16:8	Low pO_2 *vs* Amb pO_2	992	236	756
	Hypoxia *vs* Amb pO_2	2043	395	1648
	Low pO_2 *vs* Hyp	414	65	349
16:8 *vs* 8:16	Amb pO_2	3983	1428	2555
	Low pO_2	2378	963	1415
	Hypoxia	2659	939	1720
